# Effects of aerobic exercise and dietary flavonoids on cognition: a systematic review and meta-analysis

**DOI:** 10.3389/fphys.2023.1216948

**Published:** 2023-08-16

**Authors:** Daren Kumar Joseph, Arimi Fitri Mat Ludin, Farah Wahida Ibrahim, Amalina Ahmadazam, Nur Aishah Che Roos, Suzana Shahar, Nor Fadilah Rajab

**Affiliations:** ^1^ Center for Healthy Ageing and Wellness (H-CARE), Faculty of Health Sciences, Universiti Kebangsaan Malaysia, Kuala Lumpur, Malaysia; ^2^ Center for Toxicology and Health Risk Studies (CORE), Faculty of Health Sciences, Universiti Kebangsaan Malaysia, Kuala Lumpur, Malaysia; ^3^ Faculty of Medicine and Defence Health, National Defence University of Malaysia, Kuala Lumpur, Malaysia

**Keywords:** flavonoids, aerobic exercise, cognition, biomarkers, neurobehavioral assessment abbreviations

## Abstract

**Introduction:** Studies have shown that exercise increases angiogenesis and perfusion in the hippocampus, activates neurogenesis in the dentate gyrus and increases synaptic plasticity, as well as increases the complexity and number of dendritic spines, all of which promote memory function and protect against cognitive decline. Flavonoids are gaining attention as antioxidants in health promotion due to their rich phenolic content, particularly for their modulating role in the treatment of neurodegenerative diseases. Despite this, there has been no comprehensive review of cognitive improvement supplemented with flavonoid and prescribed with exercise or a combination of the two interventions has been conducted. The purpose of this review is to determine whether a combined intervention produces better results when given together than when given separately.

**Methods:** Relevant articles assessing the effect of physical exercise, flavonoid or in combination on cognitive related biomarkers and neurobehavioral assessments within the timeline of January 2011 until June 2023 were searched using three databases; PubMed, PROQUEST and SCOPUS.

**Results:** A total of 705 articles were retrieved and screened, resulting in 108 studies which are in line with the objective of the current study were included in the analysis.

**Discussion:** The selected studies have shown significant desired effect on the chosen biomarkers and neurobehavioral assessments.

**Systematic Review Registration:** identifier: [CRD42021271001].

## 1 Introduction

Cognitive decline, which is initially slow yet progressive, can be exacerbated by various risk factors and events (Legdeur et al., 2018). Among the reported cellular and molecular events that underlie cognitive decline are oxidative stress, deposition of protein aggregates, neuroinflammation, impaired mitochondrial function, induction of apoptosis, and alteration of autophagy (Wang et al., 2018). These impairments negatively impact the quality of life and daily functions of affected individuals. Pharmacological treatments or drugs used in clinical practice are often directed toward alleviating disease symptoms rather than reversing or improving neurodegeneration or reducing cognitive and functional declines. Extensive studies have been conducted to prove that physical activities have a neuroprotective effect by slowing the progression of neurodegeneration and thus improving or enhancing cognitive function. The protective effect of physical exercise increases the level of brain-derived neurotrophic factor (BDNF) and catecholamines, such as dopamine and epinephrine (Meis et al., 2017). In addition to exercise, nutrition is an important means to reduce the incidence of cognitive decline, Alzheimer’s disease (AD), and Parkinson’s disease (PD). Therefore, polyphenols derived from plant food play an important role in supporting the development and maintenance of a healthy brain (Braga et al., 2018). Polyphenols principally comprise flavonoids, and previous research indicates that regular consumption of foods containing flavonoids may lower the risk of neurodegenerative diseases (Jung and Kim, 2018). Because of their ability to cross the blood–brain barrier (BBB), flavonoids are considered a potential agent for slowing down cognitive decline (Mansour et al., 2017). The current review aims to determine the effect of physical exercise, flavonoid supplementation, and/or a combination of both interventions on biomarkers and neurobehavioral outcomes in rodent models with cognitive impairment. Existing evidence has shown that physical exercise and flavonoids have individual benefits. However, little is known about the effectiveness of the intervention when administered concurrently in an animal model. Animal models would provide critical information and understanding of the synergistic effect of flavonoids and exercise on cognition enhancement. A positive outcome would help identify lifestyle alterations affecting cognition and prevent cognitive impairment in the aging society over time.

## 2 Methods

This systematic review was conducted in accordance with the Preferred Reporting Items for Systematic Review and Meta-Analyses (PRISMA) guidelines (Page et al., 2021). We only included studies that examined cognitive decline with neurobehavioral and biomarker evaluation after administration of any amount of flavonoids, forced exercise, or both in male rodents. The meta-analysis included works that reported similar or related outcomes under similar or related experimental conditions. Other reports describing different measures or sets of experiments were also included in the review, but they were not included in the meta-analysis. The detailed protocol for this review has been registered in the PROSPERO database (Registration No: CRD42021271001).

### 2.1 Eligibility criteria

Studies using male rodents with the cognitive impairment model assessing the effects of exercise, flavonoids, or a combination of both were eligible for inclusion. The types of exercise searched were treadmill exercise, swimming training, wheel running, and rotarod exercise. Epigallocatechin-3-gallate (EGCG), grape seed proanthocyanidin extract (GSPE), 7,8-dihydroxyflavone (DHF), silibinin, quercetin, and spinosin were some of the commonly used flavonoids; they were studied and included. Comparators that were considered eligible were animals induced with aluminum chloride (AlCl_3_), scopolamine, amyloid beta (Aβ), streptozotocin (STZ), and young rats. Eligible studies were selected independent of the number of animals in the experiment, exercise speed, duration and time of training, and duration and dosage of flavonoids used. Only experimental studies were included in the review. These experimental studies must have used flavonoids, exercise, or a combination or both as interventions, and the studies must have conducted behavioral tests, biochemistry assessments, or both to determine cognitive improvement. However, studies on humans, female rodents, and non-rodent animals were excluded from this systematic review, as well as studies lacking original data (e.g., letters, review articles, or editorials), articles on unrelated topics, and non-specific exercise intervention. In this work, analysis and results were separated according to three types of interventions: aerobic exercise, flavonoids, and combined intervention. For the meta-analysis, results that allowed a pool of data were selected. When data were unavailable, the work was not included in the meta-analysis.

### 2.2 Data sources and search strategy

Comprehensive and systematic searches were conducted by an independent author using electronic databases such as PubMed, PROQUEST, and SCOPUS. The following MESH and search terms were used: [Flavonoids] AND [aerobic exercise] AND [Cognition] OR [Biomarkers]. Duplications were removed using Rayyan, a web application for systematic reviews (Ouzzani et al., 2016). Manual searches were also conducted and supplemented using reference lists from identified articles. Electronic databases were searched from January 2011 to June 2023.

### 2.3 Study selection and data extraction

The titles and abstracts of every citation in the literature search were independently screened by two (DK and AA) of the seven listed authors. Titles and abstracts clearly dealing with a different subject were excluded. All other data were extracted directly from the full-text articles, and those with potential relevance were examined for eligibility criteria. According to PRISMA recommendations, inclusion and exclusion criteria were based on relevant study characteristics (animals, intervention, comparator, outcome, and study design). Studies were included if 1) experiments involved male rodents; 2) the intervention was any form of supervised exercise; and 3) the comparator was exercise, flavonoids, or a combination of both in animal models of cognitive impairment. Any disagreement was resolved by consensus or a consultation with a third reviewer (NACR). After applying the exclusion and inclusion criteria in the selected articles, the following important data were extracted: author names, year of publication, study origin, animal model (species, age, and weight), duration of intervention, exercise protocol description, flavonoids, and dosage. The outcome data extracted comprised neurobehaviors and biomarkers assessed in the included studies. All authors involved were contacted to provide missing data or clarify if the data provided were ambiguous.

There were studies evaluating the effects of physical exercise in models of pregnancy, inflammation, ovariectomized animals, stress model, neuropathic pain, amyotrophic lateral sclerosis, intracerebral hemorrhage, smoking, encephalomyelitis, schizophrenia, and cerebral ischemia. All these findings were outside the scope of the current review and were thus excluded.

### 2.4 Risk of bias and calculations

All selected articles were evaluated according to the 10-item checklist for risk of bias of the Systematic Review Centre for Laboratory Animal Experimentation (SYRCLE). Possible biases affecting the selected articles were evaluated using SYRCLE’s Risk of Bias Tool, a screening instrument specifically developed for risk of bias assessment in animal studies (Hooijmans et al., 2014).

A “yes” score indicates a low risk of bias, a “no” score indicates a high risk of bias, and an “unclear” score indicates an unknown risk of bias. The quality of the included studies was independently evaluated by two reviewers (DK and AA). Any discrepancies were resolved by discussion or by consulting a third reviewer (NACR). A risk-of-bias summary table was created using Review Manager, version 5.3, and included in the Supplement (Figure 2).

### 2.5 Data synthesis and statistical analysis

The demographic information and quality of the included studies were described narratively and tabulated accordingly. Where appropriate, meta-analyses were conducted for data that were suitable to be pooled together using the RevMan 5.4 software.

The continuous data extracted from individual studies were pooled together using their reported mean and standard deviation (SD), with the mean difference (MD) and 95% confidence interval (CI) used as effect estimates. The pooled effect estimates were reported as MD together with its 95%CI. Heterogeneity across the included studies in a meta-analysis was assessed using the Chi-squared test and Higgin’s I^2^ test for heterogeneity. Heterogeneity is considered to be low, moderate, or high if the I^2^ test is 30%, 50%, or 75%, correspondingly. A random effects (RE) meta-analysis was performed considering the presence of heterogeneity across the included studies, with a *p*-value <0.05 indicating statistical significance. Sensitivity analysis was performed by conducting a fixed-effect (FE) model to test for result robustness. The limited number of studies available for meta-analysis did not permit a subgroup analysis. Publication bias was not evaluated because fewer than 10 studies were included in the meta-analyses conducted. The Cochrane Review (Page et al., 2019) recommended that publication bias is inappropriate when fewer than 10 studies are synthesized.

## 3 Results

### 3.1 Study selection and data extraction

**FIGURE 1:**
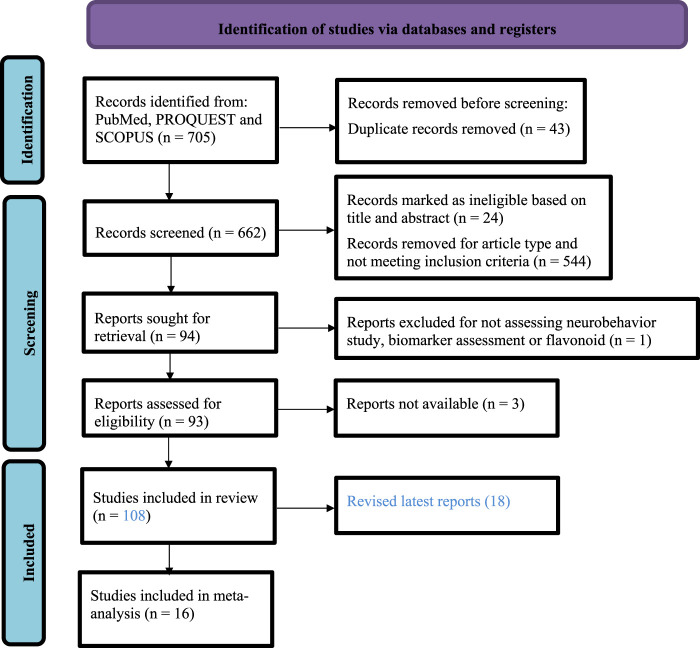
Flowchart of the publication search and selection process.

Initially, 705 articles were retrieved, of which, after thorough scrutinization, 63 were duplicated; 62 focused on other populations; 285 described other diseases; 203 were either reviews, short communication, conference abstracts, talks, or posters; 33 did not analyze the effects of exercise and flavonoids on brain alterations although cognitive impairment was reported, 44 focused on study design not related to flavonoids, exercise, or both on cognitive impairment; and 11 did not include neurobehavioral assessment or biomarkers. Finally, 108 studies that involved aerobic exercise and flavonoids and assessed neurophysiological effects on behavior and brain alterations were selected. For the meta-analysis, 16 studies with aggregate data for pooling were selected for meta-analysis on neurobehavioral tests and biomarkers. Figure 1 shows the flowchart of the data-gathering process. The included study characteristics are summarized in SupplementaryTable 1.

### 3.2 Study characteristics

The included animal species were male rodents aged between 4 weeks and 20 months. The number of animals in each group varied from 4 to 42.

#### 3.2.1 Exercise

In the experimental models of exercise, exercise was in various forms, including aerobic exercise, running wheel, swim training, and rotarod device. The training time for voluntary and forced exercise ranged from 7 days to 16 weeks. The treadmill speed ranged from 8–13.2 m/min, and the duration was 30 min/m. The exercise was continued in one study until fatigue. The positive impact of exercise on cognition is through the molecular process of improving the redox state and enhancing inflammatory defenses (Benedetto et al., 2017). This would, in turn, trigger angiogenesis and neurogenesis and improve synapse formation (Chen et al., 2019). Exercise is considered as a safe and economical approach to neuroprotection and neurorestorative in cognitive impairment (Hsueh et al., 2018).

#### 3.2.2 Flavonoids

Concerning flavonoids, the dosage ranged from 2 μg/kg to 1,650 mg/kg, and supplementation of flavonoids varied from 30 min to 9 months. The most common flavonoid supplementation protocol was 14 days (2 weeks). Among the common flavonoids found in the studies are catechin and epigallocatechin gallate (EGCG). EGCG is known to induce neurogenesis due to the ability of flavonoids and their metabolites to penetrate the blood–brain barrier and reach the brain (Unno et al., 2017). Moreover, EGCG eliminates ROS by inducing the production of proinflammatory cytokines (Wu et al., 2017). Conversely, catechin provides neuroprotection by reducing oxidative stress and decreasing lipid peroxidation, thus improving memory (Zamani et al., 2019).

### 3.3 Quality evaluation of the included studies

Studies included in this meta-analysis did not specifically describe sample-size calculation, allocation concealment, blinded assessment of outcomes, or reported animals excluded from the analysis, as is common in animal studies. Within each study, six domains were evaluated, and each was given a high, unclear, or low-risk rating. Across all the studies combined, high, unclear, or low risk was determined by the majority. All studies revealed a low risk of bias.

### 3.4 Results of individual studies

The Morris water maze (MWM) test (*n* = 71) was the most frequently used outcome measurement for neurobehavioral assessment, followed by the passive avoidance (*n* = 25), open-field (*n* = 17), Y-maze (*n* = 17), and novel object recognition (*n* = 15) test. Meanwhile, the two most commonly measured biomarkers of interest were acetylcholinesterase (AChE) (n = 24) and BDNF (n = 17), whereas malondialdehyde (MDA) (*n* = 25), SOD (*n* = 26), and CAT (*n* = 19) were the most commonly measured oxidative stress indicators in the selected studies.

Studies have reported that factors related to response to stress and neurogenesis were 24 on AChE. Reports on cerebral oxidative stress were 25 on MDA, whereas studies investigating spatial learning and memory in laboratory rats using the most common method (MWM) were 71. Regions frequently evaluated were the hippocampus, followed by the cerebral cortex. The included articles also reported the other means of assessing cognitive function, such as immunohistochemistry of vascular endothelial growth factor (VEGF)/platelet-derived growth factor (PDGF), neurological function of mice by Zea Longa scores, image analysis using light microscopy, electroencephalograph (EEG), circadian locomotor rhythm, and TUNEL assay for detecting apoptosis of hippocampal neurons. However, these methods are not within the scope of this review.

#### 3.4.1 Morris water maze

Studies reported on various neurobehavioral tests, such as the radial maze test, Barnes hole-board maze, novel object recognition test, T-maze, open-field test (OFT), Y-maze test, and MWM test*.* The MWM test is employed to estimate memory and spatial learning in rodents, which assesses animals on time spent in the target quadrant, escape latency, and mean swimming speed. Fifty-nine studies reported on MWM, suggesting it is one of the most used neurobehavioral tests. Gibbons et al. (2014) administered a combined intervention of both flavonoids and exercise (epicatechin and β-alanine and voluntary wheel running). However, they did not observe any independent or additive/synergistic effects of the EGCG/β-ala diet on the performance in the MWM, whereas exercise improved age-related reductions in behavioral performance. This is because the dosages of EGCG and β-ala used in the study may have been too low. Conversely, Zhang et al. (2016) used combined intervention (epicatechin and treadmill exercise), indicating that treadmill exercise only improved spatial learning deficits rather than memory impairment. However, the combination therapy was able to improve both spatial learning and memory activity. Concerning flavonoid intervention, 75 studies reported its effect on learning and memory, of which only one study (Giacomini et al., 2019) showed that 40 days of 7,8-dihydroxyflavone had no sign of improvement in spatial learning and memory. It must be noted that the tests used to evaluate cognitive performance, such as contextual fear conditioning, novel object recognition, and MWM tests, may exhibit different sensitivity to treatment. Nevertheless, all other 74 studies using flavonoids reported a significant reduction in the MDA levels. These results indicated that flavonoids reduced the concentration of MDA to protect the antioxidant system.

#### 3.4.2 Malondialdehyde

Thirty-eight studies reported several factors and variables related to oxidative stress; for example, superoxide dismutase (SOD) activity, catalase and glutathione (GSH) activity, nitric oxide synthase (iNOS), and lipid peroxidation (MDA) were evaluated. Nineteen studies reported on MDA. The combined study of flavonoids with treadmill exercise by Zhang et al. (2016) reported that impaired antioxidant enzymes were restored with epicatechin treatment alone. The possible explanation is that treadmill exercise may have elevated serum corticosterone levels similar to mild stress, which might offset the anti-oxidative effect of epicatechin in the combination group. However, the combined study of flavonoids with aerobic exercise by Abhijit et al. (2018) showed that adult rats benefited more from a combination of exercise and flavonoid supplementation than a single intervention. Similarly, all 24 studies with the intervention of flavonoids alone showed that the MDA level decreased in animals. Chronic stress can stimulate oxidative stress and the increased production of free radicals, which may contribute to cognition impairment. The outcomes of the studies show that flavonoids may inhibit neurological damage by suppression of oxidative stress and further protect against learning and memory impairments.

#### 3.4.3 Acetylcholinesterase

Of 24 studies, 23 evaluated the content of AChE in rodents treated with flavonoids and one evaluated that content with a combination of flavonoids and exercise. The study using the combination of flavonoids and exercise (*n* = 44) reported that single interventions failed to show any significant decreases in AChE activity, unlike the combined interventions that resulted in a significant decrease. Among the 23 studies involving flavonoids, Mundugaru et al. (2017) reported no statistically significant changes (n = 24). However, they revealed a reduction, although the changes were not statistically significant compared to the AlCl_3_-alone group. All other studies showed a significant reduction in the level of AChE with the duration of flavonoid administration ranging from 30 min before sacrifice for AChE activity assay to 60 days. AChE is essential in maintaining the normal function of the nervous system, and an increased AChE level causes cognitive dysfunction. In the studies examined, it can be concluded that both administration of flavonoids alone and in combination with exercise could ameliorate learning and memory impairments by inhibiting AChE activity and elevating the level of neurotransmitter ACh in the cortex and hippocampus.

### 3.5 Meta-analysis

#### 3.5.1 Morris water maze

**FIGURE 2:**
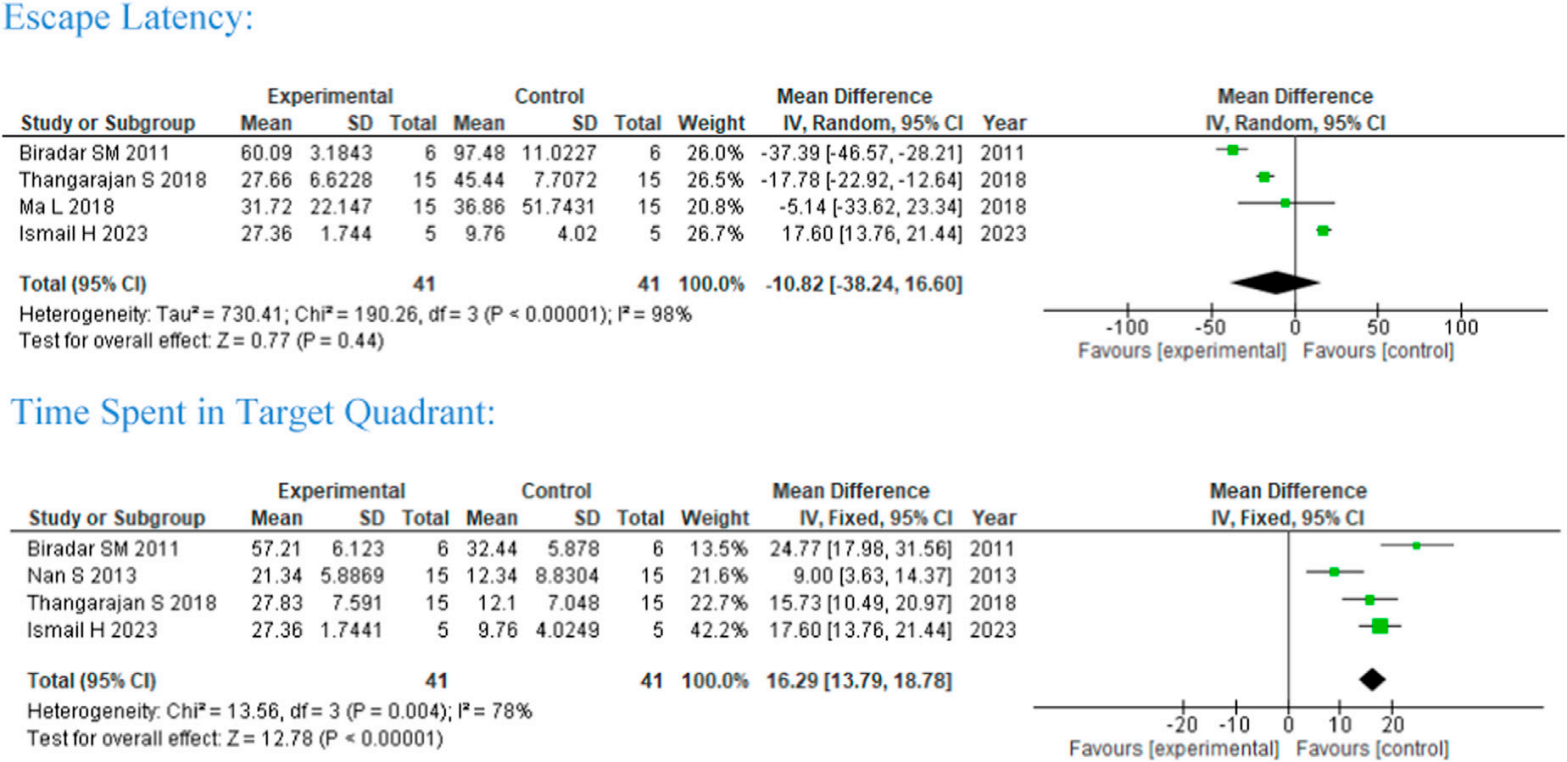
Forest plot of flavonoid treatment on MWM.

Five studies were included in the meta-analysis on the effect of flavonoid treatment on the neurobehavioral study of MWM (rodents, *n* = 41) (Figure 2). One subgroup analysis was performed with flavonoid intervention between 2 and 8 weeks. Hippocampal spatial perception and learning are commonly assessed using MWM through escape latency and time spent in the target quadrant. Among the studies, a 2-week intervention of Morin and *Vitis vinifera* (Ma et al., 2018; Thangarajan et al., 2018) and a 15-day intervention of *Ageratum conyzoides* (Biradar and Joshi, 2011) favors experimental/intervention group shortened escape latency and stronger learning ability, but they did not reach significance.

While 8-week intervention of EGCG (Nan et al., 2021) shows that studies conducted by Nan et al. (2013), Biradar and Joshi (2011), Thangarajan et al. (2018), and Ismail et al. (2023) favor control significantly for time spent at the target quadrant, implying memory was not improved (four studies, MD = 16.29, 95%CI 13.79, 18.78) with a heterogenicity of 78% between studies.

#### 3.5.2 Malondialdehyde

**FIGURE 3:**
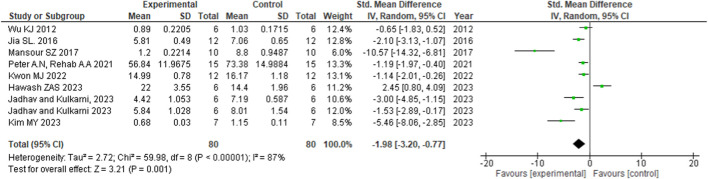
Forest plot of flavonoid treatment on MDA.

The meta-analysis focused on assessing the effect of flavonoid treatment on the oxidative stress marker of MDA (rodents, *n* = 80) (Figure 3). The nine studies included in this meta-analysis examined the effect of flavonoids on the MDA level. Brain cells are most susceptible to lipid peroxidation, which is indicated by the presence of MDA in tissues. A lower level of MDA indicates reduced oxidative stress and an improved antioxidant system in the brain tissue. This reflects on better cognitive function. The statistical results demonstrated that flavonoid administration [nine studies; mean difference (MD), −1.98 nmol/mg protein; 95%CI (−3.20, −0.77)] has a significant effect in reducing MDA level among the animals with cognitive impairment as the effect favors experimental/intervention group, with a heterogenicity of 87% between studies. The nine studies included are as follows: Wu et al. (2012) administered green tea extract (GTex), epigallocatechin gallate (EGCG), for 7 days; Jia et al. (2016) administered liquiritin for 2 weeks; Mansour et al. (2017) administered 5, 7-dihydroxyflavone for 3 weeks; Peter AN and Rehab AA 2021 administered hesperidin for 8 weeks; Kwon et al. (2022) administered *Rhodiola sachalinensis* for 3 weeks; Hawash et al. (2023) administered jambolan fruit ethanolic extract for 28 days; Jadhav and Kulkarni (2023) administered baicalein for 21 days and quercetin for 42 days, respectively; and finally, Kim et al. (2023) administered *Sesamum indicum* L. for 4 weeks.

#### 3.5.3 Acetylcholinesterase

**FIGURE 4:**
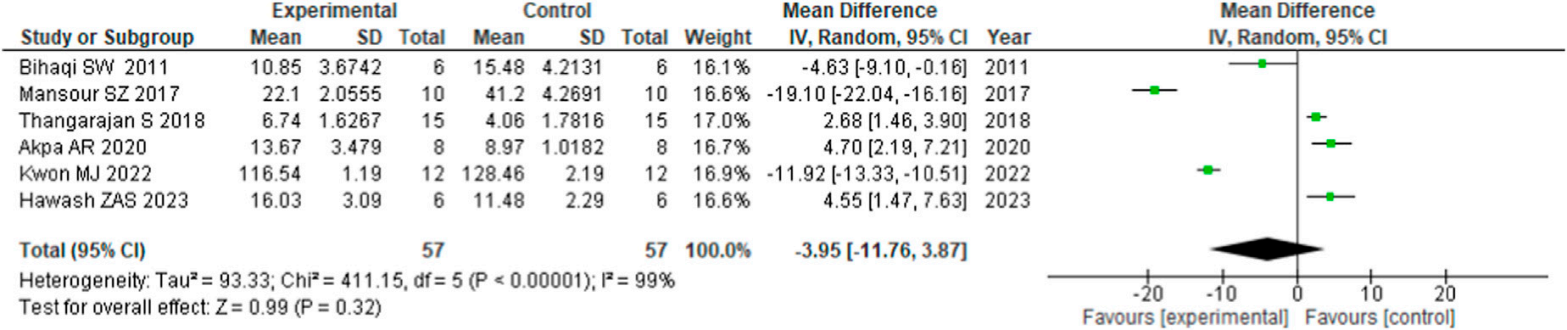
Forest plot of flavonoid treatment on AChE.

Finally, the meta-analysis studied the effect of flavonoids on AChE secretion (rodents, *n* = 57) (Figure 4). The forest plot of the pooled level of AChE for each study comparison is presented in Figure 4. Compared with the control group, *Convolvulus pluricaulis* was administered for 7 days (Bihaqi et al., 2011), 5, 7-dihydroxyflavone and *Rhodiola sachalinensis* were administered for 3 weeks, respectively (Mansour et al., 2017; Kwon et al., 2022) favors experimental/intervention group, which had positive effects on cognitive-enhancing activities from the inhibition of the AChE activity. Administration of flavonoids resulted in the improvement of the AchE activity, which invariably enhanced the neuromuscular activity. This showed that flavonoids have AChE restoration properties. However, other studies [i.e., Thangarajan et al. (2018) administering Morin for 2 weeks; Akpa et al. (2020) and Hawash et al. (2023) administering fisetin for 7 weeks and *Convolvulus pluricaulis* 1 week, respectively] showed a negative effect, favoring control. In addition, six studies [mean difference (MD), −3.95 U/mg protein; 95%CI (−11.76, 3.87)] found a trend in flavonoids improving cognition through AChE but did not reach significance, with a heterogenicity of 99% between studies.

95%CI values are presented for individual studies as squares and lines and meta-analysis results as diamonds.

## 4 Discussion

Physical exercise and flavonoids as a non-conventional approach/intervention for medical disease have proven beneficial in reducing the risk for many diseases, including stroke, high blood pressure, and mental disorders, such as chronic stress and depression (da Silva et al., 2012; Singh et al., 2014). Although the impact of physical exercise and flavonoid supplementation on cognition is well understood, the combined effect of the two interventions is still in its infancy (Wang et al., 2022). Neurodegeneration in the brain is progressive in nature and is an irreversible process. There is no effective treatment for age-related memory impairment, emphasizing the importance of developing preventive strategies before or during aging (Souza et al., 2015). Exercise has been shown to decrease the levels of circulating inflammatory cytokines, whereas flavonoids have been shown to exert beneficial effects in a variety of bodily functions and organs, including the brain (El-Kader and Al-Shreef, 2018; Nkpaa and Onyeso, 2018). The mechanisms by which flavonoids exert their effects depend largely on their antioxidant properties. Flavonoids may also interact with neuronal receptors and kinase signaling pathways, thereby modulating certain cellular processes.

The objective of the current study was to conduct a systematic review and meta-analysis to identify and evaluate the scientific literature published on the effect of exercise and flavonoid intervention on cognitive impairment, either alone or in combination. A total of 83 studies (*n* = 3,658–3,823) investigated the effect of flavonoid treatment on cognitive impairment. Five studies (Mundugaru et al., 2017; da Silveira et al., 2016; Peter and Rehab, 2021; Giacomini et al., 2019; Mallien et al., 2019) that administered flavonoids to rodents with cognitive impairment found no significant effect. A non-significant effect was observed in behavioral studies, which is the hallmark of learning, memory, and motor function. This could result from tests used to evaluate cognitive performance with different sensitivity to treatment in terms of dosage and duration. One study showed no difference in oxidative stress markers of SOD and CAT activities. This is most likely because the antioxidant activities of flavonoids may involve enzymatic and non-enzymatic pathways, such as GSH. However, the results of the remaining studies predominantly showed cognitive improvement. Elevated MDA means elevated lipid peroxidation and increased oxidative stress. Increase in lipid peroxidation of nueronal membrane leads to neuronal damage and apoptosis (Park et al., 2018). Dietary antioxidants enhance antioxidative systems and administration of phenolic-rich compounds, inhibiting MDA production (You et al., 2020). Thus, biomarker selection is critical to accurately reflect the actual scenario. Acetylcholine is one of the most important neurotransmitters involved in cognitive function regulation, and flavonoids appear to improve learning/acquisition and memory retention by lowering AChE levels. Acetylcholine modulates synaptic plasticity via BDNF. BDNF is crucial in supporting the survival and function of existing nerve cells while promoting the growth and differentiation of new neurons and synapses. As a result, BDNF is critical in learning, memory, and motor function.

Although improved behavioral performance is interpreted as improved cognition in terms of learning and memory, Chen et al. (2019) investigated the effect of treadmill exercise on cognitive impairment. The neurobehavioral radial maze test revealed that treadmill exercise for 7 or 14 days improves motor and cognitive functions. This demonstrates that exercise can improve cerebrovascular and neuronal plasticity, thereby protecting the brain from cognitive dysfunction and neurodegenerative diseases. Enhanced BDNF levels were also observed from treadmill training because BDNF is involved in the brain plasticity processes associated with cognitive recovery. Finally, six studies reported combined interventions of flavonoids and exercise. However, Bhattacharya et al. (2015) found no significant influence on learning and memory measures when the interventions were combined. Two studies (Abhijit et al., 2017; Abhijit et al., 2018) examining the combined effect showed that flavonoids alone improved the neurobehavioral test or biomarker. However, the combined intervention did not show further improvement. Two other studies (Gibbons et al., 2014; Zhang et al., 2016) found that exercise alone improved the results. Nevertheless, Ramis et al. (2021) reported that the combined action of exercise and a polyphenol-enriched diet could be very useful as a therapy to delay or ameliorate the cognitive and motor decline associated with aging by improving monoaminergic neurotransmitters. In summary, the outcomes of the studies that combine physical exercise and dietary flavonoids are varied. Additionally, the number of studies with combined intervention is limited in this review. Thus, further studies are required to properly deduce whether a physically active lifestyle in combination with the intake of antioxidants can be the most effective management strategy to alleviate the cognitive and motor deterioration associated with aging.

## 5 Strength and limitations

Although our search was comprehensive, we may have overlooked potentially relevant studies published in a language other than English. Moreover, the studies available for the meta-analysis were few. However, the strength of this study lies in its comprehensive assessment of this topic.

## 6 Conclusion

Considering the findings of this study, as well as the limitations, it can be concluded that combined intervention of exercise and flavonoids suggests a positive effect on cognitive function compared to flavonoids and exercise alone. Nevertheless, the results yielded from this review should be interpreted with caution due to the high heterogeneity observed across the included studies. Therefore, further research is necessary to define more specific recommendations on these interventions, in terms of quantity and type of polyphenol, as well as exercise strategies, to recommend these interventions as part of a healthy lifestyle regime in humans.

## Data Availability

The original contributions presented in the study are included in the article/Supplementary Material; further inquiries can be directed to the corresponding author.

## Abbreviations

BDNF, brain-derived neurotrophic factor; MWM, Morris water maze; AChE, acetylcholinesterase; MDA, malondialdehyde; EGCG, epigallocatechin-3-gallate; PAT, passive avoidance test.
