# Corrigendum: Effects of aerobic exercise and dietary flavonoids on cognition: a systematic review and meta-analysis

**DOI:** 10.3389/fphys.2023.1287170

**Published:** 2023-09-25

**Authors:** Daren Kumar Joseph, Arimi Fitri Mat Ludin, Farah Wahida Ibrahim, Amalina Ahmadazam, Nur Aishah Che Roos, Suzana Shahar, Nor Fadilah Rajab

**Affiliations:** ^1^ Center for Healthy Ageing and Wellness (H-CARE), Faculty of Health Sciences, Universiti Kebangsaan Malaysia, Kuala Lumpur, Malaysia; ^2^ Center for Toxicology and Health Risk Studies (CORE), Faculty of Health Sciences, Universiti Kebangsaan Malaysia, Kuala Lumpur, Malaysia; ^3^ Faculty of Medicine and Defence Health, National Defence University of Malaysia, Kuala Lumpur, Malaysia

**Keywords:** flavonoids, aerobic exercise, cognition, biomarkers, neurobehavioral assessment abbreviations

In the published article, there was an error with the **affiliation** numbers. Instead of “Daren Kumar Joseph^1^, Arimi Fitri Mat Ludin^1^, Farah Wahida Ibrahim^2^, Amalina Ahmadazam^1^, Nur Aishah Che Roos^3^, Suzana Shahar^1^, Nor Fadilah Rajab^2^*”, it should be “Daren Kumar Joseph^1^, Arimi Fitri Mat Ludin^1^, Farah Wahida Ibrahim^2^, Amalina Ahmadazam^1^, Nur Aishah Che Roos^3^, Suzana Shahar^1^, Nor Fadilah Rajab^1^*”.

The authors apologize for this error and state that this does not change the scientific conclusions of the article in any way. The original article has been updated.

