# Supplementary material for: Effects of aerobic exercise and dietary flavonoids on cognition: a systematic review and meta-analysis
Source: Front Physiol. 2023 Aug 16;14:1216948. doi: 10.3389/fphys.2023.1216948 (PMC10468597; doi:10.3389/fphys.2023.1216948)
Supplement: Supplementary file 1 [file Table1.docx]

**Supplementary Table 1:** Characteristics of the included studies.

| **Exercise** | **No** | **Author,**  **Origin** | **Animal model** | **Age of Animal** | **Duration of intervention** | **Intervention** | **Comparator** | **Outcome measured** | | **Improvement on learning and memory** |
| --- | --- | --- | --- | --- | --- | --- | --- | --- | --- | --- |
|  |  |  |  |  |  |  |  | **Neurobeha-vioral Assessment** | **Biomarker** |  |
|  | 1 | Chen Z et al. 2019, China | Mice, C57BL/6 | NA | 7 or 14 days | treadmill exercise - 8–10 m/min for 30 min/day and continued 5 days/week |  | Radial Maze Test | BDNF | Improved |
|  | 2 | Xu et al. 2022, China | APP/PS1 transgenic mice | 3 months | 12 weeks | Treadmill – 5 m/min for 5 min, 8 m/min for 5 min (warming  up), 12 m/  min for 30 min (main exercise), and 12 m/  min for 5 min (recovery) for 5 days per week for 12 weeks |  | MWM |  | Improved |
| **Exercise and Flavonoid** | 3 | Abhijit S et al. 2017, India | Rats, Wistar | 4 & 18 months | 120 days | 400 mg/kg/day grape seed proanthocyanidinextract (GSPE)  Swimming Training - 2 h/day and 5 days/week with an intensity of 3% of their body weight tied to their tails as steel washers | sedentary controls (n=11)  vs  swimming trained (n=11)  vs  swimming trained and supplemented with GSPE (n=11)  vs control supplemented with GSPE (n=11) | T-maze | AChE activity | Improved |
|  | 4 | Abhijit S et al. 2018, India | Rats, Wistar | 4 & 18 months | 16 weeks | 400 mg/kg/day grape seed proanthocyanidin extract (GSPE) | sedentary controls  vs  sedentary supplemented with GSPE  vs  swimming trained supplemented with GSPE |  | lipid peroxidation (LPO)  GSH  Glutathione peroxidase (GPx) activity  CAT activity  GR activity | Improved |
|  | 5 | Bhattacharya TK. et al. 2015, USA | Mice, BALB/cJ | 10 weeks | 39 days | 1.49 mg of EGCG per gram of diet  3.34 mg of B-ALA per gram of diet  23 cm diameter running wheel | Control (n=11 sedentary and 11 runner)  vs  B-ALA (n=11 sedentary and 12 runner)  vs  EGCG (n=12 sedentary and 11 runner)  vs  EGCG and B-ALA (n=12 sedentary and 11 runner) | Contextual fear conditioning  Cued fear conditioning |  | No Improvement |
|  | 6  **Exercise and Flavonoid** | Gibbons TE. et al. 2015, USA | BALB/cByJ mice | 19 months | 28 days | mice ingested on average 182 mg/kg/day and 417 mg/kg/day, of EGCG and β-ala, respectively.  housed in cages with access to a running wheel | Sedentary mice (n=12)  vs  Standard diet (n=14)  vs  VWR mice (n=15)  vs  EGCG and β-ala diet (n=15) | Morris Water Maze  Contextual Fear Conditioning | BDNF | Improved |
|  | 7 | Ramis MR et al. 2021, Spain | Rats, Wistar | 18 months | 28 days of catechin  4 weeks rotarod | 20 and 40 mg/kg  Catechin  5 days/week  Rotarod | Control group (n=7)  vs  exercise group (n=7)  vs  rotarod exercise+catechin (n=7) | Radial Maze Test  Barnes Holeboard Maze  Novel Object Recognition Test | Tryptophan Hydroxylase (TPH)  Tyrosine Hydroxylase (TH) | Improved |
|  | 8  **Exercise and Flavonoid** | Zhang Z et al. 2016, China | Mice, APP/PS1 | 8 months | 4 months | 50 mg/kg  Epicatechin  30 min a day, 13.2 m/min for 5 days/week  Treadmill exercise | nTg group (n=8)  vs  Untreated‑Tg (n=8)  vs  epicatechin‑Tg group (n=8)  vs  treadmill exercise‑Tg group (n=8)  vs  COMA group (n=8) | Morris water maze test | GSSG  SOD  GSH  LPO  CAT  BDNF expressions  Aβ deposition | Morris water maze test – Improved by Exercise alone    GSSG, SOD, GSH, LPO, CAT – Improved by Ep alone  BDNF & Aβ – Improved by combination |
| **Flavonoid**  **Flavonoid** | 9 | Kwon et al. 2022, Korea | ICR mice | 8 weeks old | 3 weeks | 50, 100, and 200 mg/kg/day  Tyrosol-Enriched Rhodiola sachalinensis  Extracts | Normal (n=12)  vs  Control (n=12)  vs  Bio-RSE 50 (n=12)  vs  Bio-RSE 100 (n=12)  vs  Bio-RSE 200 (n=12)  vs  DNPZ (n=12) | Y maze  MWM  PAT | MDA  AChE | Improved |
|  | 10 | Ali et al. 2023, Egypt | Wistar Rat | 6 weeks old | 4 weeks | 50 and 100 mg/kg  *Yucca aloifolia L*. extract | group 1- normal control group (n=8)  vs  group 2- rotenone (n=8)  vs  group 3 and 4-  Yucca extract (50 and 100 mg/kg) (n=8) | Rotarod Test  OFT  Forced Stress Swim Test | LPO  GSH  NO  SOD | Improved |
|  | 11 | Hawash et al. 2023, Egypt | Sprague-Dawley |  | 28 days | 500 mg/kg bw  Jambolan fruit ethanolic extract | control group (n=6)  vs  group 2 - Alzheimer’s disease (n=6)  vs  group 3 -  jambolan fruit (n=6)  vs  group 4 -Rivastigmine (n=6)  vs  group 5 - choline chloride (n=6)  vs  group  6 - jambolan fruit and choline (n=6) | Morris water maze | MDA  NO  CAT  SOD | Improved |
|  | 12 | Kim et al. 2023, Korea | ICR mice | 6 weeks | 4 weeks | 250 mg/kg  500 mg/kg  *Sesamum indicum* L. | group 1- normal (n=7)  vs  group 2- control (scopolamine) (n=7)  vs  group 3- positive control (donepezil) (n=7)  vs  group 4 GBE-250 & group 5 GBE-500 (Goenback extract) (n=7)  vs  group 6 GBO-1 & group 7 GBO-2 (Goenback oil) (n=7)  vs  group 8, M74-  250 & group 9, M74-500 (M74 extract) (n=7)  vs  group 10 M74-1& group 11 M74-2, (M74 oil) (n=7) | PAT  Morris Water Maze | BDNF  AChE | Improved |
|  | 13 | Zhong et al. 2023, China | P301s-tau transgenic mouse  C57BL/6 | 6 months old | 3 months | 10 µg/mL  Quercetagetin drugs | C57BL/6 mice (N=7–10)  vs  P301S-tau transgenic mice (N=7–10)  vs  P301S-tau transgenic mice + quercetagitrin (N=7–10) | Y-maze  Morris Water Maze |  | Improved |
|  | 14 | Lee et al. 2023, Korea | C57BL/6 | 8 weeks old | 12 days | 100 mg/kg  The *gastrodia elata‘s* rhizome extract (GER)  The *glycyrrhiza uralensis*‘s radix (GUR)  mixture of GER and GUR 7:3 = GGW73. | group 1, control  vs  group 2, scopolamine (SCP)  vs  group 3, SCP + GER  vs  group 4, SCP + GUR  vs  group 5, SCP + GGW73 10 mg/kg  vs  group 6, SCP + GGW73 100 mg/kg  vs  group  7, SCP + GGW73 200 mg/kg  vs  group 8, SCP + Donepez | Y-maze  PAT  Morris Water Maze | BDNF | Improved |
|  | 15 | Jadhav and Kulkarni. 2023, India | Wistar rat | not mentioned | 21 days | 10 mg/kg  Baicalein treatment | group 1 normal control (n=6)  vs  group 2 disease control (n=6)  vs  group 3 memantine (n=6)  vs  group 4 baicalein (n=6)  vs  group 5 memantine and baicalein (20 + 5 mg/kg) & group 6 memantine  and baicalein (20 + 10 mg/kg) (n=6) | MWM  Elevated Plus Maze  PAT | MDA  SOD  GSH  AChE  BDNF | Improved |
|  | 16 | Jadhav and Kulkarni. 2023, India | Wistar rats | not mentioned | 42 days | 25 and 50 mg/kg  Quercetin and Memantine treatment | normal control group (n=10)  vs  disease control group (n=10)  vs  memantine group (n=10)  vs  group quercetin (n=10)  vs  group memantine  and quercetin 25 mg/kg (n=10)  vs  memantine and quercetin 50 mg/kg (n=10) | MWM  EPM  PAT | MDA  AChE  BDNF | Improved |
|  | 17 | Mani et al. 2023, Saudi Arabia | Sprague-dawley | 3 months old | 30 days | Methanolic Ajwa Seed Extract treatment (MASE) | control group I (n=6)  vs  LPS-induced group II (n=6)  Vs  groups III and IV  MASE 200 mg/kg or 400 mg/kg (n=6) | Elevated Plus Maze  NORT | ACh | Improved |
|  | 18 | Ismail et al. 2023, Pakistan | Sprague-Dawley rats |  | 21 days | 100mg/kg, 200mg/kg,  and 400mg/kg  *Phoenix dactylifera* extract | Normal control (n=5)  vs  diabetes control (n=5)  vs  diabetes-positive control  Glibenclamide (n=5)  vs  AD control (n=5)  vs  Alzheimer’s positive control  (Rivastigmine) (n=5)  vs  Phoenix dactylifera seed extract groups (n=5) | Elevated Plus Maze  Morris Water Maze | ACh | Improved |
|  | 19 | Lee et al. 2023, Korea | Sprague-Dawley | 7 weeks old | not mentioned | *Chrysanthemum boreale* (Makino) extract | Normal (n=10)  vs  NC (Sco) (n=10)  vs  CB extract-low (CBL) (n=10)  vs  CB extract-high (CBH) (n=10)  vs  Donepezil (positive control) (n=10) | PAT | BDNF | Improved |
|  | 20 | Alaqeel et al. 2022, Saudi Arabia | Albino rats | not mentioned | 140 ±10g | 15 mg/kg  Quercetin Nanoemulsion | negative control group (I) (n=8)  vs  QC group (n=8)  vs  Alzheimer’s group (AD) (n=8)  vs  treated group  (I): AlCl3+QC free  nanoemulsion (n=8)  vs  treated group (II): AlCl3+QC (n=8)  vs  treated group (III): AlCl3+QCNE (n=8) |  | SOD  GSH  MDA  AOPP | Improved |
|  | 21 | Yeh et al. 2022, Taiwan | C57BL/6 | 8 weeks | 10 days | 25 and 50 mg/kg  Tea Seed Kaempferol Triglycoside (KXRG) | group 1, control (n=8)  vs  group 2, LPS (n=8)  vs  group 3, LPS+25 mg/kg KXRG (n=8)  vs  group 4, LPS+50 mg/kg KXRG (n=8) | Y-maze | SOD | Improved |
|  | 22 | Pang et al. 2022, Korea | ICR mice | 5 weeks | 14 days | 50 and 100 mg/kg/  day  *Cirsium japonicum var*. Maackii extract | sham group (n=7)  vs  control group (n=7)  vs  ECJM50 group (n=7)  vs  ECJM100 (n=7)  vs  donepezil group (n=7) | T maze  NORT  MWM | MDA  NO | Improved |
|  | 23 | Kang et al. 2022, China | APP/PS1 and  C57BL/6J | 6 months | 70 days | 1000 mg/kg/d  Tieguanyin Extracts | control (10 mice, C57BL/6J)  vs  model (8 mice, APP/PS1)  vs  Donepezil (8 mice, APP/PS1)  vs  Tgy-Q (8 mice, APP/PS1, 1000 mg/kg/d),  vs  Tgy-N (8 mice, APP/PS1, 1000 mg/kg/d)  vs  Tgy-C (8 mice, APP/PS1, 1000 mg/kg/d) | MWM  NORT | MDA  NF-κB p65 TNF-α  IL-1β  IL-6 | Improved |
|  | 24 | Jajin EM et al. 2021, Iran | Rats, Wistar | 7 weeks | 42 days | 25 mg/kg/day  SPION,  Quercetin,  QT-SPION respectively | control group (n=8)  vs  sham group (n=8)  vs  AlCl3 group (n=8)  vs  AlCl3 + SPION group (n=8)  vs  AlCl3 + QT group (n=8)  vs  AlCl3 + SPION-QT group (n=8) | Morris Water Maze Test  Passive Avoidance Test | AChE | Improved |
|  | 25 | Kim CJ et al. 2021, Korea | Mice, C57BL | 9 weeks | 7 weeks | 125, 250, and 500 mg/k  HLJG0701 | control group (n=10)  vs  donepezil group (n=10)  vs  scopolamine-induced control group (n=10)  vs  HLJG0701- groups (n=10) | Morris Water Maze Test  Y-Maze Task | AChE  ACh  MDA  CAT | Improved |
|  | 26 | Nan S et al. 2013, China | Rats, Sprague-Dawley | NA | 8 weeks | Low dose: 100 mg/kg/d  Moderate dose: 250 mg/kg/d  High dose: 625 mg/kg/d  EGCG | sham (n=15)  vs  AD group (n=15)  vs  AD + EGCG groups (n=15) | Morris Water Maze Test | GPx  T-SOD  MDA | Improved |
|  | 27 | Wen H 2020, China | Rats, Sprague-Dawley | NA | 30 days | 50 mg/kg  Black chokeberry | sham (n=12)  vs  Aβ group (n=12)  vs  Aβ + SMB CAN group (n=12) | Morris Water Maze Test | DPPH  ABTS | Improved |
|  | 28 | Zhang Y et al. 2019, China | Rats, Sprague-Dawley | NA | 28 days | 16.5 and 33 mg/kg  Sailuotong | control group (n=8)  vs  model group (n=8)  vs  SLT- group (n=8) | Morris Water Maze Test | IL-6  IL-12  IL-1α chemokine CXCL10 | Improved |
|  | 29  **Flavonoid** | Long Jy et al. 2020, China | Rats, Sprague-Dawley | 4–6 weeks | 3 days | low dose - 25 mg/kg  middle dose - 50 mg/kg  high dose - 100 mg/kg  Naringin | control group (n=11)  vs  sham group (n=11)  vs  positive control group (n=11)  vs  model group (n=11)  vs  naringin group (n=11) | Morris Water Maze Test  New object recognition test (NORT) | MDA  SOD  GSH-Px | Improved |
|  | 30  **Flavonoid** | Kim MJ et al. 2019, Korea | Mice, ICR | 4-week | 3 weeks | 50 and 100 mg/kg  Green tea seed oil (GTO) | NC group (n=13)  vs  Aβ group (n=13)  vs  GTO group (n=13) | Y-Maze Test  Passive Avoidance Test  Morris Water Maze Test | MDA  SOD  GSH | Improved |
|  | 31 | Wang YJ et al. 2019, China | Mice, Kunming | 6-week | 30 days | 100, 200, and 400 mg/kg  Centipeda minima | vehicle control (n=10)  vs  D-gal/AlCl3 + vitamin E (n=10)  vs  D-gal/AlCl3 group (n=10)  vs  D − gal/AlCl3 + ECM (n=10) | Morris Water Maze Test | MDA  GSH  SOD | Improved |
|  | 32 | Sethiya NK et al. 2019, India | Rats, Sprague-Dawley rats | 2-3 months | 14 days | 400 mg/kg  Shankhpushpi extract | Group I - vehicle (distilled water) only (n=6)  vs  Group II - scopolamine (n=6)  vs  Group III - piracetam (n=6)  vs  Group IV-VII - CD, CT, CP and EA. (n=6) | Cook and Weidley's pole climbing apparatus  Morris Water Maze Test | AchE | Improved |
|  | 33 | Doungue HT et al. 2018, Cameroon | Rats, Wistar rats | 3-month | 60 days/ 2 months | 200 and 400 mg/ kg bw  Passiflora edulis | Group (NC): normal (n=10)  vs  Group VE400: AD + Vitamin E (n=10)  vs  Group (PC): AD-induced rats (n=10) vs  Group (A.E200) & Group (A.E400) (n=10) | Morris Water Maze Test | AchE | Improved |
|  | 34 | Sun K et al. 2018, China | mice, ICR | 8 week | 6 weeks | 2 mg/kg and 10 mg/kg  Matrine | control group (n=9)  vs  VE group(n=9)  vs  D-gal group (n=9)  vs  MAT low-dose group and MAT high-dose group | NOR  Y-Maze Test  Weight-Loaded Swimming Test | IL-1β  IL-6  T-SOD  MDA | Improved |
|  | 35  **Flavonoid** | Ma L. et al. 2018, China | Rats, Sprague-Dawley | 7–8 weeks | 14 days | Low dose: 50 mg/kg  Medium dose: 150 mg/kg  High dose: 300 mg/kg  Vitis vinifera | control group (n=15)  vs  donepezil (n=15)  vs  model group (n=15)  vs  low-dose, medium-dose, high-dose VTF groups (n=15) | Morris water maze task | AChE  ChAT | Improved |
|  | 36  **Flavonoid** | Mostafa N M 2018, Egypt | Rats, Sprague-Dawley |  | 1 week | 200 mg/kg/day  Bombax ceiba | Group 1: Control group (n=6)  vs  Group 2: distilled water + scopolamine (n=6)  vs  Group 3: donepezil (n=6)  vs  Group 4: BCLE (n=6) | Morris Water Maze Test  Passive Avoidance Test | Catalase MDA  AChE Activity | Improved |
|  | 37 | Chen Z J et al. 2018, China | mice, SAMP8 and SAMR1 | three months | 9 months | 50 mg/ kg/ day  Psoralea corylifolia | normal control group SAMR1 (n=10)  vs  SAMP8-RSV group (n=10)  vs  model control group (n=10)  vs  SAMP8-TPFB group (n=10) | Morris Water Maze Test | TNF-α,  IL-1β,  IL-6  level of Aβ- 42 | Improved |
|  | 38 | Mundugaru R et al. 2017, India | mice, Swiss albino |  | 45 days | 200 and 400 mg/kg body weight  Pluchea lanceolata | Group 1: vehicle control (n=6)  vs  Group 2: AlCl3 (n=6)  vs  Group 3 and 4: HAPL (n=6) | Forced swim test  Tail suspension test | AchE  catalase,  lipid peroxidation,  glutathione peroxidase | Forced swim test-improved  Tail suspension test & Biomarkers – no improvement |
|  | 39  **Flavonoid** | Halawany AME et al. 2017, Egypt | mice, Swiss albino |  | 7 days | 10 and 20 mg/kg/day  Gingerol | Group 1: saline (n=10)  vs  Group 2: STZ (n=10)  vs  Group A: STZ with celecoxib (n=10)  vs  Group B & C: STZ with gingerol (n=10) | Morris Water Maze Test  Y-maze | measurement of amyloid β-42  COX-2 deposition | Improved |
|  | 40 | Bhatnagar M et al. 2017, India | mice, BALB/c | 10-14 weeks | 21 days | 40 mg/kg of body weight  Centella asiatica and/or Withania somnifera | No Herbal Ext  vs  Centella Ext  vs  Withania Ext  vs  Withania & Centella Ext  vs  MPTP Centella Ext  vs  MPTP Withania Ext  vs  MPTP No Herbal Ext  vs  MPTP Withania & Centella Ext | Akinesia  Catalepsy  Swim-test | SOD  CAT  Gpx  GSH  LPO | Improved |
|  | 41 | Choi SY et al. 2017, Korea | mice, ICR | 5 weeks | 14 days | 100 and 200 mg/kg/day  Acer okamotoanum | normal group (n=4)  vs  control group - Ab25–35 (n=4)  vs  EA groups (n=4) | Morris water maze test  Novel object recognition test  T-maze test | reduced nitric oxide  AChE activity | Improved |
|  | 42  **Flavonoid** | Koh EJ et al. 2017, Korea | mice, ICR | 5 weeks | 4 weeks | 200 and 400 mg/kg body weight/day  Spirulina maxima | control (n=6)  vs  donepezil (n=6)  vs  scopolamine group (n=6)  SM70EE groups (n=6) | Passive Avoidance Test  Morris Water Maze Test | AChE  GSH  GPx | Improved |
|  | 43 | Thangthaeng N et al. 2016, USA | Rats, Fischer 344 | 19 months | 7 weeks | 2% (w/w)  Montmorency tart cherry | Control (n=12)  vs  Tart cherry group (n=12) | Morris Water Maze Test | NOX-2, COX-2 | Improved |
|  | 44 | Prema A et al. 2016, India | Rats, Wistar | 10-12 weeks | 6 weeks | 2.5%, 5% & 10%  fenugreek seed powder | Group I - control (n=6)  vs  Group II - AlCl3 (n=6)  vs  Group III - V (n=6)  vs  Group VI - 10% FSP (n=6) | Passive avoidance test  Morris Water Maze Test | AChE | Improved |
|  | 45  **Flavonoid** | Silveira CCSM et al. 2016, Brazil | Rats, Wistar | Three month | 30 min before the assays | 1, 3, and 30mg/kg  ethanolic extract of the yellow propolis (EEYP) | control group (n=10)  vs  caffeine group (n=10)  vs  EEYP groups (n=10) | Open Field (OF) Test  Elevated Plus Maze (EPM) Test  Forced Swimming (FS) Test  Step-Down Inhibitory Avoidance (IA) Test | MDA  CAT  SOD | FS showed no improvement  All other behavior test and biomarkers showed improvement |
|  | 46 | Lee HY et al. 2016, Rep of Korea | mice, ICR | 5 weeks | 4 days | 200 and 400mg/kg  Aronia melanocarpa berries | control group (n=7)  vs  positive control group (donepezil) (n=7)  vs  scopolamine group (n=7)  vs  A. melanocarpa berries groups (n=7) | Morris Water Maze Test  Passive Avoidance Test | AChE activity | Improved |
|  | 47 | Kim MS et al. 2014, Korea | mice, C57BL/6 | 8 weeks | 14 days | 100 and 200mg/kg  Fructus mume extract | vehicle + vehicle (n=10)  vs  F. mume (n=10)  vs  donepezil + scopolamine (n=10)  vs  vehicle + scopolamine (n=10)  vs  F. mume + scopolamine (n=10) | Morris Water Maze Test | AChE Activity  ChAT Expression | Improved |
|  | 48  **Flavonoid** | Zhang Y et al. 2014, China | mice, Chinese Kunming | 6 to 8 weeks | 12 days | 100, 200 and 400 mg/kg  Ziziphi Spinosae Semen | Control (n=12)  vs  piacetam (n=12)  vs  model group (n=12)  vs  WES groups (n=12) | Y‑maze test  Passive avoidance test |  | Improved |
|  | 49 | Baitharu I et al. 2014, India | Rats, Sprague-Dawley |  | 28 days | 10 mmol kg-1 day-1  Withania somnifera | Normoxia (n=15)  vs  Normoxia + Withanolide A (n=15)  vs  Hypoxia + Vehicle (n=15)  vs  Hypoxia + Withanolide A (n=15) | Morris Water Maze Test | GSH  LPO | Improved |
|  | 50 | Weon JB et al. 2014, Korea | mice, ICR | 4-week | 120 min | 10, 50, and 100mg/kg  Loranthus parasiticus | Control group (n=7)  vs  Donepezil group (n=7)  vs  5scopolamine treated group (n=7)  vs  Loranthus parasiticus groups (n=7) | Morris Water Maze Test  Passive Avoidance Test |  | Improved |
|  | 51 | George A et al. 2014, Malaysia | mice, C57BL/6 | 2-6 months | 14 days | 50 mg/kg  Gingko biloba  50 and 100 mg/kg Polygonum minus | Vehichle (n=12–14)  vs  Donepezil (n=12–14)  vs  Vehicle + Scopolamine (n=12–14)  G. biloba and the P. minus treated groups (n=12–14) | Barnes maze test |  | Improved |
|  | 52  **Flavonoid** | Hong SW et al. 2013, Rep of Korea | Rats, Sprague Dawley | 8 weeks | 1 week | 5, 10, and 20 mg·kg-1  soyasaponin I | sham group (n=12)  vs  IBO group (n=12)  vs  soya-I groups (n=5-6) | Y-Maze task  Passive avoidance task  Morris Water Maze Test |  | Improved |
|  | 53 | Zhao L et al. 2013, China | mice, APP/PS1 double transgenic | 4 months | 12 weeks | 40 mg/kg  Apigenin | WT control (n=4)  vs  WT + apigenin (n=5)  vs  APP/PS1 control (n=5)  vs  APP/PS1 + apigenin (n=5) | Morris Water Maze Test | Aβ Levels BDNF  SOD  GSH-Px | Improved |
|  | 54 | Weon JB et al. 2013, Rep of Korea | mice, ICR | 3 weeks | 90 min | 100, 300 and 500 mg/kg  Codonopsis lanceolata | Control (n=7)  vs  Donepezil (n=7)  vs  Scopolamine (n=7)  vs  Scopolamine + C. lanceolata (n=7)  vs  Scopolamine + fermented C. lanceolata (n=7) | Morris Water Maze Test  Passive Avoidance Test |  | Improved |
|  | 55  **Flavonoid** | Wu KJ et al. 2012, Taiwan | Rats, Sprague–Dawley | 8-9 weeks | 7 days | 30, 100, and 300 mg/kg  green tea extract  10 mg/kg  epigallocatechin gallate | sham group (n=6)  vs  pentoxifylline group (n=6)  vs  ischemia/reperfusion induction group (n=6)  vs  GTex groups (n=6)  vs  EGCG group (n=6) | Morris Water Maze Test | MDA  SOD  GSH  COX-2  iNOS | Improved |
|  | 56 | Cong WH et al. 2012, China | mice, transgenic APP/PS1 | 5 months | 3 months | 44mg/kg/day  WNK(Wei Nao Kang) consisting of P. ginseng, G. biloba, and saffron. | nontransgenic littermates + tap water (n=10)  vs  EGB761 30 mg/kg/day (n=10)  vs  APP/PS1+tap water (n=9)  vs  APP/PS1 + WNK (n=10) | Morris Water Maze Test | Aβ-level | Improved |
|  | 57 | Bihaqi SW et al. 2011, India | rats, Wistar |  | 7 days | 100, 150, and 250 mg/kg  Convolvulus pluricaulis | Group I - control (n=6)  vs  Group II - scopolamine (n=6)  vs  Groups III, IV, and V - AE (n=6) | Elevated Plus Maze  Morris Water Maze Test | AChE | Improved |
|  | 58  **Flavonoid** | Biradar SM et al. 2011, India | mice, Swiss albino | 3-4 months | 15 days | 250, 500, 750 mg/kg  Ageratum conyzoides | Normal control - NaCl (n=6)  vs  Piracetam group (n=6)  vs  Vehicle control group (n=6)  vs  Scopolamine control (n=6)  vs  ACEt+scop (n=6) | Elevated plus maze  Morris Water Maze Test | AChE | Improved |
|  | 59 | Zhoa Q et al 2011 | Mice, SAMP8 and SAMR1 | 6 weeks | 38 days | 750 mg/kg  Chotosan | sham-operated controls (n=9-10)  vs  T2VO control (n=9-10)  vs  T2VO + CTS (n=9-10) | Elevated plus maze test  Nobel object recognition test (ORT)  Morris Water Maze Test | VEGF | Improved |
|  | 60 | Peter A.N, Rehab A.A 2021, Egypt  **Flavonoid** | rats, Sprague-Dawley | 8–10 weeks | 8 weeks | 100 mg/kg  Hesperidin | Group 1 (control group) (n=15)  vs  Group 2 (EMB group) (n=15)  vs  Group 3 (HSP group) (n=15)  vs  Group 4 (EMB + HSP group) (n=15) | Forced swim test  Y-maze test  Rod walking test | CAT  GSH  MDA  TNF-α  IL-1β  BDNF | Rod walking test, TNF-α, IL-1β – significant improvement  Forced swim test, Y-maze test, CAT, GSH & MDA, BDNF levels – no significant improvement |
|  | 61 | Oyovwi M O et al 2021, Nigeria | rats, Wistar | 6-8 weeks | 28 days | 20 mg/kg/day  Quercetin | group 1 - normal (n=5)  vs  group 2 - corn oil (n=5)  vs  group 3 - quercetin (n=5)  vs  groups 4 – endosulfan (n=5)  vs  group 5 - quercetin + endosulfan (n=5) |  | MDA  GPx  SOD  CAT  AChE | Improved |
|  | 62 | Imran I et al. 2021, Pakistan | Rats, Sprague-Dawley |  | 41 days | 5, 10, 20, and 30% dilutions (v/v in tap water)  Grewia asiatica  **Flavonoid** | Group 1 - control (n=6-8)  vs  Groups 2 to 5 - G. asiatica exudate + Scopolamine (n=6-8)  vs  Groups 6C - Scopolamine + piracetam (n=6)  vs  Groups 7 -Scopolamine (n=6) | Open Field Test  Elevated Plus Maze Test  Forced Swimming Test  Passive Avoidance Test  Y-Maze Test  Novel Object Recognition Test  Morris Water Maze Test | AChE  MDA  SOD  GPx | Improved |
|  | 63 | Li S et al. 2020, China | Rats, Wistar |  | 8 weeks | 100, 200 mg/(kg·bw)/day – rutin  100, 200 mg/(kg·bw)/day – puerarin  100, 200 mg/(kg·bw)/day - silymarin | control (n=10)  vs  AlCl3-toxicity (n=10)  vs  rutin, puerarin, silymarin (n=10) | Morris Water Maze Test | SOD  GSH-Px MDA | Improved |
|  | 64 | Kim J et al. 2020, Korea | mice, CD-1 | 6 weeks | 1 hr | 0.3, 1 or 3 mg/kg casticin | control group (n=6)  vs  donepezil (n=6)  vs  scopolamine-treated group (n=6)  vs  V. rotundifolia & casticin (n=6) | Passive avoidance test  Novel object recognition test  Morris water maze test | AChE  BDNF | Improved |
|  | 65  **Flavonoid** | Kim HJ et al. 2020, Korea | mice, ICR | 6 weeks | 14 days | 10, 20 and 30 mg/kg  Ribes diacanthum Pall (RDP) | Control (n=6)  vs  Tacrine group (n=6)  vs  Scopolamine group (n=6)  vs  RDP10; RDP20; RDP30 groups (n=6) | Morris Water Maze Test  Passive Avoidance Test |  | Improved |
|  | 66 | Das J et al. 2020, India | Rats, Wistar | young (6-7 months), aged (18-20 months) | 3 weeks | 20 mg/kg body wt/day  Fisetin | Young control (n=10)  vs  Young fisetin supplemented (n=10)  vs  Aged control (n=10)  vs  Aged fisetin supplemented (n=10) | MWM test  Open-field test | Lipid Peroxidation | Improved |
|  | 67 | Gao WL et al. 2020, China | Rats, Sprague-Dawley |  | 7 weeks | 50, 100 and 200 mg/kg  Grape seed proanthocyanidin extract (GSPE) | control group (n=9)  vs  STZ model group (n=9)  vs  STZ plus GSPE groups (n=9) | Morris Water Maze Test | MDA  SOD  GSH | Improved |
|  | 68 | Massaquoi MS et al. 2020, USA | mice, C57BL/6J | 8 weeks | 8 weeks | 5 mg/kg  7,8-dihydroxyflavone (DHF) | VEH group (n=17)  vs  VEH+DHF group (n=18)  MPTP group (n=19)  vs  MPTP+DHF group (n=20) | Grip test analysis | tyrosine hydroxylase (TH) | Improved |
|  | 69  **Flavonoid** | Keser H et al. 2020, Turkey | mice, C57BL/6 | young (5 months) old (18 months) | 3 weeks | 5 mg/kg  7,8-dihydroxyflavone (DHF) | young control (n=10)  vs  old control (n=10)  vs  old-DHF (n=7) | Hang wire  Adhesive removal | MDA  SOD  GSH | Improved |
|  | 70 | Liu P et al. 2020, China | Rats, Sprague–Dawley |  | 30 days | 25, 50 and 100 mg/kg  Silibinin | control group (n=8-10)  vs  STZ-injected plus donepezil-treated group (n=8-10)  vs  STZ-model group (n=8-10)  vs  STZ-injected plus silibinin-treated group (n=8-10) | Open field test  Y maze test  Novel objects recognition test  Morris water maze test |  | Improved |
|  | 71 | Wang GW et al. 2019, China | Rats, Sprague-Dawley | aged rats (21.90±0.22 months); young rats (10 weeks) | 30 days | 25, 50 and 100 mg/kg  Bidens pilosa L. (BP) | young group (n=10)  vs  aged control group (n=10)  vs  EEBP treated aged group (n=10) | Open-field task  Passive avoidance task  Morris water maze test |  | Improved |
|  | 72 | El-Gazar AA et al. 2019, Egypt | Rats, Sprague-Dawley | 7 days | 7 days | 10 mg/kg  Morin | group 1 - normal control (n=19)  vs  group 2 - mTBI control group (n=19)  vs  groups 3 and 4 - mRTBI-5 & 7 (n=19)  vs  groups 5 and 6 - DMSO (vehicle) (n=19)  vs  group 7 - Morin treated group (n=19)  vs  group 8 - MK-801 treated (n=19)  vs  group 9 - Morin and MK-801 (n=19) | Swimming test  Conditioned – Avoidance (CA) test | inflammatory mediators - TNF-α, IL-6, p-NF-κB p65  apoptotic/anti-apoptotic markers - caspase-3 & Bcl-2  dementia markers - Aβ42 & p(thr231)Tau | Improved |
|  | 73  **Flavonoid** | Liu B et al. 2019, China | Rats, Sprague–Dawley | 8–10 weeks | 45 days | 25, 50, and 100 mg/kg  Silibinin | sedentary group (n=10)  vs  OT plus resveratrol-treated group (n=10)  vs  overtraining (OT) group (n=10)  vs  OT plus silibinin-treated groups (n=10) | Morris Water Maze Test  Novel Object Recognition Test | MDA  T-SOD  CAT | Improved |
|  | 74 | Pyrzanowska J et al. 2019, Poland | Rats, Sprague-Dawley | 9 months | 10 weeks | 1:100 n=8), 2:100 (n=8) and 4:100 (n=9)  Aspalathus linearis | control (n=8)  vs  A. linearis infusion pre-treated groups | Morris Water Maze Test | Dopamine level | Improved |
|  | 75 | Giacomini A et al. 2019, Italy | Mice, Ts65Dn | 4 months | 39 days | 5.0 mg/kg | Euploid+7,8-DHF (n=7)  vs  Euploid+Vehicle (n=7)  vs  Ts65Dn+Vehicle (n=6)  vs  7,8-DHF treated group (n=6) | Morris Water Maze Test | levels of TrkB | No improvement |
|  | 76 | Bax EN et al. 2019, USA | Mice, C57Bl6J | 5 months | 1 month | 10 mg/kg body weight | HFD + vehicle (n=7)  vs  HFD + S-equol (n=7) | Elevated plus maze  Tail suspension test |  | Improved |
|  | 77 | Chen X et al. 2019, China | Rats, Sprague-Dawley |  | 7 days | 2 μg/kg wt  Astragaloside VI | sham control group (n=6)  vs  MCAO group (n=6)  vs  Astragaloside VI group (n=6) | Step-through passive avoidance task  Morris Water Maze Test | MDA,  protein carbonyl ROS | Improved |
|  | 78  **Flavonoid** | Zhuang J et al. 2019, China | Mice, ICR | 7 weeks | 20 weeks | 100 mg/kg  Purple sweet potato color (PSPC) | control group (n=10)  vs  PSPC group (n=10)  vs  HFD group (n=10)  vs  HFD + PSPC group (n=10) | Step-through passive avoidance task  Morris Water Maze Test | BDNF | Improved |
|  | 79 | Sun P et al. 2019 | Rats, Sprague Dawley | 8 weeks | 21 days | 100 and 200 mg/kg  Dihydromyricetin (DHM) | Sham group (n=10)  vs  AD group (n=10)  vs  AD + DHM groups (n=10) | Morris Water Maze Test | IL-1β  IL-6  TNF-α  Bcl-2  Bax | Improved |
|  | 80 | Mallien AS et al. 2019, Germany | Mice, C57BL/6 N Crl | 8 weeks | 21 weeks | soy-containing animal diet | standard diet group (n=12)  vs  soy-free group (n=12) | Open field–novel object test  Elevated O-maze test  Dark–light test  Novel object recognition test.  Fear conditioning  Social memory test  Forced swim test  Hot-plate test  Puzzle box test |  | No improvement |
|  | 81  **Flavonoid** | Khan A et al. 2018, South Korea | Mice, C57BL/6N | 8 weeks | 2 week | 30 mg/kg/day  Quercetin | Control mice (n=15)  vs  Mice injected with LPS (n=15)  vs  Mice injected with LPS and quercetin (n=15) | Y-Maze Test  Morris Water Maze Test | TNF-a  COX-2  NOS-2  Bax/Bcl-2 | Improved |
|  | 82 | He L et al. 2018, China | Rats, Sprague-Dawley |  | 20 days | 4, 8, and 16 mg/kg  Icariside II (ICS II) | Sham (n=10)  vs  Sham+ICS II-H (n=12)  vs  IBO+donepezil (n=14)  vs  Model (IBO) (n=15)  vs  IBO+ICS II-L, IBO+ICS II-M, and IBO+ICS II-H (n=14) | Morris water maze test |  | Improved |
|  | 83  **Flavonoid** | Thangarajan S et al. 2018, India | Rats, Wistar |  | 14 days | 40 mg/kg b.wt.  Morin | Group I (Control) (n=15)  vs  Group II (PbAc) (n=15)  vs  Group III (PbAc+Morin) (n=15)  vs  Group IV (Morin alone) (n=15) | Rotarod activity  Morris water maze test  Open field test  Forced swim test  String test for grip strength  Adhesive removal test | SOD  CAT  GPx  GR, glutathione-S-transferase  reduced glutathione  AChE  LPO  NO  protein carbonyls | String test for grip strength – no improvement |
|  | 84 | Mansour SZ et al. 2017, Egypt | Rats, Wistar |  | 21 days | 50 mg/kg b.wt.  5, 7-dihydroxyflavone | Group 1 (Control) (n=10)  vs  Group 2 (ACR) (n=10)  vs  Group 3 (DHF) (n=10)  vs  Group 4 (IR) (n=10)  vs  Group 5 (DHF+ ACR) (n=10)  vs  Group 6 (DHF+ IR) (n=10) |  | MDA  AChE  caspase-3  BDNF | Improved |
|  | 85  **Flavonoid** | Parashar A et al. 2017, India | Mice, Swiss albino | 7–8 months | 21 days | 100 mg/kg  Rutin | group I: control + vehicle (n=8)  vs  group II:control + rutin (n=8)  vs  group III: CUS + vehicle (n=8)  vs  group IV: CUS+ rutin (n=8) | Open field  Beam walk  Elevated plus maze  Novel object recognition test |  | Elevated plus maze - no improvement |
|  | 86 | Gong Y et al. 2017, China | Rats, Sprague-Dawley | 35–38 days | 34 days | 1 and10 mg/kg  hyperoside | control group (n=12)  vs  fluoxetine group (n=12)  vs  vehicle (equal volume of DMSO) group (n=12)  vs  hyperoside groups (n=12) | Morris water maze test  forced swim test | corticosterone  BDNF | Improved |
|  | 87 | Ay M et al. 2017, USA | Mice, MitoPark transgenic & C57BL6-LC | 12 weeks | 8 weeks | 25 and 175 mg/kg  Quercetin | vehicle (n=8 or 9)  vs  quercetin groups (n=8 or 9) | open-field apparatus  rotarod test |  | Improved |
|  | 88 | Liu W et al. 2017, China | Mice, C57BL/6 | 10 weeks | 1 week | 183, 550 and 1650 mg/kg  Peganum harmala Linn | vehicle-treated group (n=10)  vs  scopolamine-induced group (n=10)  vs  EXT-treated groups (n=10) | Morris water maze test | AChE and ACh levels  choline acetyltransferase (ChAT) activity | Improved |
|  | 89  **Flavonoid** | Chen Q and Hub P, 2017, China | Rats, Sprague-Dawley |  | 8 weeks | 12.5, 25, and 50 mg/kg  proanthocyanidins (PAC) | control group (n=8)  vs  PAC group (n=8)  vs  ethanol group (n=8)  vs  ethanol and PAC groups (n=8) | Morris water maze test | TBARS  GSH  IL-1β and TNF-α | Improved |
|  | 90 | Lee Y et al. 2016, Korea | Mice, ICR | 6 weeks | 14 days | 1.25, 2.5, 5 and 10 mg/kg  Spinosin | vehicle-treated control (n=9-10)  vs  piracetam-treated control (n=9-10)  vs  Spinosin treated groups (n=9-10) | Passive avoidance task | BDNF | Improved |
|  | 91 | Lee HE et al. 2016, Rep of Korea | Mice, ICR | 6 weeks | 1 hr before acquisition | 1.25, 2.5, 5 and 10 mg/kg  Swertisin | control group (n=8–10)  vs  donepezil group (n=8–10)  vs  scopolamine group (n=8–10)  vs  Swertisin group (n=8–10) | Passive avoidance task  Morris water maze test  Y-maze task  Open field test |  | Passive avoidance task, Morris water maze task – Improved  Y-maze task, Open field test – No improvement |
|  | 92 | Ortiz-Lopez L et al.2016, Mexico | Mice, Balb/C | 8–10 weeks | 14 days | 0.625, 1.25, 2.5, 5.0 and 10 mg/kg body weight | control group (n=4–5)  vs  EGCG groups (n=4–5) |  | Akt protein | Improved |
|  | 93  **Flavonoid** | Li E. et al. 2016, China | Rats, Wistar |  | 8 – 11 days | 2 μg  Apelin-13 | control (n=10)  vs  Vehicle group (n=9)  vs  Imipramine group (n=9–10)  vs  Apelin group (n=7–9)  vs  Ex 1: FS group (n=10)  vs  Ex 2: FS+apelin group (n=9)  vs  Ex 3: Apelin group (n=9–10)  vs  Ex 4: LY294002 + Apelin group (n=7–9) | Learned helplessness (LH) test  Novel object recognition (NOR) test  Foot shock sensitivity test | Corticosterone assay | Learned helplessness (LH) test,  Novel object recognition (NOR) test – Improved  Foot shock sensitivity test, Corticosterone assay - No improvement |
|  | 94  **Flavonoid** | Jia SL. et al. 2016, China | Rats, Sprague–Dawley | 3 months | 14 days | 25, 50 and 100 mg/kg  Liquiritin | sham control (n=12)  vs  donepezil (Aβ1–42 + 3 mg/kg donepezil) (n=12)  vs  model (Aβ1–42) (n=12)  vs  LQ-H, LQ-M, and LQ-L groups (n=12) | Morris water maze test | GSH-Px, SOD  MDA  protein carbonyl | Improved |
|  | 95 | Wang S. et al. 2016, China | Mice, Kunming |  | 3 weeks | 20 and 40 mg/kg  Orientin | control group (n=14)  vs  orientin-H group (n=14)  vs  noise group (n=14)  vs  noise + orientin-L & H groups (n=14) | Morris water maze test  Open-field test  Step-through test | corticosterone (CORT), norepinephrine (NE) dopamine (DA) levels  MDA  SOD  GSH  CAT  BDNF | Improved |
|  | 96 | Souza LC. et al. 2015, Brazil | Mice, Swiss Albino | 3 months | 60 days | 1 and 10 mg/kg  Chrysin | Young/Vehicle (n=8–10)  vs  Aged/Vehicle (n=8–10)  vs  Aged/Chrysin groups (n=8–10) | Open-field test (OFT)  Morris water maze test | BDNF  SOD  CAT  GPx | OFT - No improvement |
|  | 97 | Moreno-Ulloa AM. et al. 2014, Brazil | Mice, C57BL/6 | 6 and 26 months | 2 weeks | 1 mg/kg of body weight  epicatechin (Epi) | Group one, Y mice (6-month-old) (n=5)  vs  group two-Senile (26-month-old) (n=5)  vs  group three-S mice treated with Epi (n=5) |  | GSH  GSSG  Catalase Activity  Protein Carbonylation | Improved |
|  | 98  **Flavonoid** | Subramanian P. et al. 2015, India | Rats, Wistar |  | 8 weeks | 50 mg/kg b.w.  Fisetin | group I – untreated control (n=6)  vs  group II – treated with fisetin (n=6)  vs  group III – injected with AC (n=6)  vs  group IV – administered with AC and fisetin (n=6) |  | Lipid hydroperoxideSOD  CAT  GSH  NO level | Improved |
|  | 99 | Li R. et al. 2014, China | Rats, Wistar |  | 26 days | 30 and 100 mg/kg  Chrysin (CH) | Control group (n=10)  vs  Vehicle group (DM) (n=10)  vs  CH groups (n=10) | Morris water maze test | MDA  CAT  SOD  GSH  TNF-a  IL-1b IL-6 levels  caspase-3 activity | Improved |
|  | 100 | Jung IH. et al. 2014, Korea | Mice, ICR | 6 weeks | 1 hr | 2.5, 5, 10 and 20 mg/kg  Spinosin | control group (n=8–10)  vs  donepezil group (n=8–10)  vs  vehicle-treated control (n=8–10)  vs  spinosin groups (n=8–10) | Passive avoidance task  Y-maze task  Morris water maze test  Open field test |  | Improved |
|  | 101  **Flavonoid** | Nassiri-Asl MN. et al. 2013, Iran | Rats, Wistar |  | 16 days | 25, 50, and 100 mg/kg  Quercetin | control group (n=10)  vs  PTZ control (n=10)  vs  Quercetin groups (n=10) | Passive avoidance apparatus | TBARS measurement  Total sulfhydryl groups assay | Passive avoidance apparatus – Improved  TBARS measurement, Total sulfhydryl groups assay – no improvement |
|  | 102 | Wang Y. et al. 2012, China | Mice, C57BL/6 | 2 months | 60 days | 20 mg/kg  Epigallocatechin-3-gallate (EGCG) | Group I - control (n=5–6)  vs  Group II - EGCG (n=5–6)  vs  Groups III EGCG + cyclopamine (n=5–6)  vs  Group IV EGCG + 2-hydropropyl-cyclodextrin (n=5–6) | Morris water maze test |  | Improved |
|  | 103  **Flavonoid** | Zhang L et al. 2020, China | Rats, Sprague–Dawley |  | 14 days | 10, 30 and 100 mg/kg/day  Eriodictyol | Vehicle Group (n=10)  vs  Fluoxetine group (n=9)  vs  LPS group (n=10)  vs  30 mg/kg eriodictyol+  LPS group (n=9)  vs  100 mg/kg eriodictyol+  LPS group (n=10). | Open field test  Forced swim test  Novel object recognition |  | Improved |
|  | 104 | Ben-Azu B et al. 2018, Nigeria | Mice, Swiss | 5–6 weeks | 30 minutes | 2.5, 5 and 10 mg/kg  Naringin | group 1 - control (n=6)  vs  groups 2-4 - NARIN (n=6)  vs  group 5 - Diazepam (n=6) | hole-board apparatus  Elevated-plus maze test  tail suspension test  forced swim test  novelty-induced rearing  open field test  light and dark box test  social interaction test  Y-maze test |  | Improved |
|  | 105  **Flavonoid** | Das J et al. 2017, India | Rats, Wistar | 3 months | 12 weeks | 20 mg/kg  Fisetin | Group I - sham controls (n=5)  vs  Group II - iron-induced epileptic rats (n=5)  vs  Group III - vehicle (DMSO) (n=5)  vs  Group IV - pre-treated iron induced rats (n=5) | Morris water maze test  Open-field test | MDA | Improved |
|  | 106 | Akpa AR et al. 2020, Nigeria | Mice, albino |  | 7 weeks | 15 mg/kg  Fisetin | Group 1(S/OIL): control (n=8)  vs  Group 2 (CPF): Chlorpyrifos (n=8)  vs  Group 3 (FIS): fsetin (n=8)  vs  Group 4: FIS + CPF (n=8) | forepaw grip | MDA  GPx  SOD  CAT  AChE | Improved |
|  | 107 | Wei P et al. 2022, China | C57 mice |  | 3 weeks | 100 and 200 mg/kg  Silibinin, | sham group (n=5)  vs  FA 2mM group (n=5)  vs  FA 2mM+ silibinin 100 mg/kg group (n=5)  vs  FA2mM+ silibinin 200 mg/kg group (n=5)  vs  memantine group (n=5) | Y -Maze  MWM | GSH | Improved |
|  | 108 | Soubh AA et al. 2021, Egypt | Sprague-Dawley (SD) rat |  | 7 days | 10 mg/kg  Morin | normal control  vs  mTBI control group  vs  groups 3 and 4  (killed 24 hrs later and after 1 week)  vs  group 5 – Morin  vs  group 6 - MK-801  vs  group 7 - Morin + MK-801 | Swimming test  Fear-Conditioned (FC) test | caspase-3 Bcl-2 | Improved |
