# Supplementary material for: Effects of aerobic exercise and dietary flavonoids on cognition: a systematic review and meta-analysis
Source: Front Physiol. 2023 Aug 16;14:1216948. doi: 10.3389/fphys.2023.1216948 (PMC10468597; doi:10.3389/fphys.2023.1216948)
Supplement: Supplementary file 2 [file DataSheet1.docx]

Appendix:
